# *Entamoeba histolytica* Adaption to Auranofin: A Phenotypic and Multi-Omics Characterization

**DOI:** 10.3390/antiox10081240

**Published:** 2021-08-02

**Authors:** Yana Shaulov, Lotem Sarid, Meirav Trebicz-Geffen, Serge Ankri

**Affiliations:** Department of Molecular Microbiology, Ruth and Bruce Rappaport Faculty of Medicine, Technion, 31096 Haifa, Israel; yana11me@campus.technion.ac.il (Y.S.); Lotemsarid@campus.technion.ac.il (L.S.); meiravg@technion.ac.il (M.T.-G.)

**Keywords:** *Entamoeba histolytica*, auranofin, drug resistance, transcriptomics, redoxomics, thioredoxin reductase

## Abstract

Auranofin (AF), an antirheumatic agent, targets mammalian thioredoxin reductase (TrxR), an important enzyme controlling redox homeostasis. AF is also highly effective against a diversity of pathogenic bacteria and protozoan parasites. Here, we report on the resistance of the parasite *Entamoeba histolytica* to 2 µM of AF that was acquired by gradual exposure of the parasite to an increasing amount of the drug. AF-adapted *E. histolytica* trophozoites (AFAT) have impaired growth and cytopathic activity, and are more sensitive to oxidative stress (OS), nitrosative stress (NS), and metronidazole (MNZ) than wild type (WT) trophozoites. Integrated transcriptomics and redoxomics analyses showed that many upregulated genes in AFAT, including genes encoding for dehydrogenase and cytoskeletal proteins, have their product oxidized in wild type trophozoites exposed to AF (acute AF trophozoites) but not in AFAT. We also showed that the level of reactive oxygen species (ROS) and oxidized proteins (OXs) in AFAT is lower than that in acute AF trophozoites. Overexpression of *E. histolytica* TrxR (EhTrxR) did not protect the parasite against AF, which suggests that EhTrxR is not central to the mechanism of adaptation to AF.

## 1. Introduction

The protozoan parasite *Entamoeba histolytica* is the etiologic agent of amoebiasis, a significant hazard in countries with low socioeconomic status and poor sanitation. This disease accounted for 55,500 deaths and 2.237 million disability-adjusted life years in 2010 [1]. The main symptoms of amoebiasis are inflammation of the large intestine and liver abscesses. Infection occurs following the ingestion of food contaminated with cysts. Trophozoites that emerge from the cysts migrate to the large intestine. Asymptomatic colonization occurs in most cases (90% of all infections). Symptomatic infection is characterized by bloody diarrhea. Metronidazole (MNZ) is the drug currently used for invasive amoebiasis [2]. Inside the parasite, MNZ is reduced through the action of thioredoxin reductase (TrxR) to a nitro radical anion or to a nitroimidazole. This nitro group reduces O_2_, leading to the formation of cytotoxic reactive oxygen species (ROS) inside the parasite. The nitroimidazole can also modify cysteine containing proteins such as thioredoxin (Trx), leading to their inactivation [3]. There are numerous common side effects related to MNZ, including dizziness, heartburn, stomach cramps, trouble sleeping, and weight loss [4,5,6]. Treatment with MNZ is usually highly effective, but resistance to this drug has been reported in various bacteria [7,8] and protozoan parasites [9,10,11]. To address these drawbacks, new alternatives to MNZ have been initiated and AF has emerged as one of the most potent anti-protozoan parasites drugs. Initially, AF was a gold-containing compound developed in the 1970s for the treatment of rheumatoid arthritis [12]. Its mechanism of action as an antiarthritic gold drug remained controversial but it is assumed that it works by inhibiting the activity of TrxR, a crucial enzyme involved in the maintenance of the redox homeostasis in the cell [13]. AF is also a potent anticancer agent [14] and has been found to be very efficient against a number of pathogens, including *Mycobacterium abscessus* [15], *Clostridium difficile* [16,17], vancomycin-resistant enterococci [18,19], and some additional multidrug resistant bacteria [20]. Auranofin is also very efficient against parasites, including the trematode *Schistosoma mansoni* [21,22], and protozoan parasites, including *Trichomonas vaginalis* [23], *Giardia lamblia* [24], and *E. histolytica* [25]. The mode of action of AF in protozoan parasites is not completely understood although it is assumed that TrxR is the main target of AF in *E. histolytica* [24,25]. In *G. lamblia*, this mechanism of action has been challenged by the significant TrxR activity that occurs in trophozoites exposed to high concentrations of auranofin [26]. Overexpression of TrxR in *G. lamblia* has no effect on the sensitivity of this parasite to AF [26]. AF can also target *E. histolytica* adenosine 5′-phosphosulfate kinase (EhAPSK), an essential enzyme in Entamoeba sulfolipid metabolism [27]. We recently showed that AF induced the formation of more than 500 oxidized proteins (OXs) in *E. histolytica*, including some crucial enzymes for redox homeostasis and cytoskeletal proteins, which are essential for *E. histolytica*’s cytoskeleton dependent virulence [28]. Knowledge about resistance to AF in bacteria and in protozoa is scarce. Recently, toxoplasma trophozoites resistant to AF (2 µM) were successfully generated through chemical mutagenesis. The authors identified point mutations in genes encoding redox-relevant proteins, such as superoxide dismutase and ribonucleotide reductase. However, recapitulation of these mutations in the parasite did not confer resistance to AF, suggesting that the mechanism of resistance is complex [29]. In this work, we used a multi-omics approach to characterize an *E. histolytica* strain that was made resistant to AF (AFAT) by progressively adapting the parasite to 2 µM of AF. At this concentration, the drug is lethal to non-adapted parasites [25,28].

## 2. Materials and Methods

### 2.1. E. histolytica Culture

*E. histolytica* trophozoites, the HM-1:IMSS strain (a kind gift of Prof. Samudrala Gourinath, Jawaharlal Nehru University, New Delhi, India), were grown and harvested according to a previously reported protocol [30].

### 2.2. Adaptation of E. histolytica Trophozoites to AF 

The concentration of AF in *E. histolytica* trophozoite culture was progressively increased from 0 to 2 µM over a period of one month.

### 2.3. Growth Rate of WT Trophozoites and AFAT

The growth rate of WT trophozoites or AFAT and their viability were measured according to a previously reported protocol [31].

### 2.4. Viability of AFAT Exposed to H_2_O_2_, Paraquat, MNZ or GSNO

The viability of WT trophozoites and AFAT exposed to H_2_O_2_ (2.5 mM for 30 min), paraquat (2.5 mM for 24 h), MNZ (5 µM for 24 h), or GSNO (350 µM for 2 h) (Sigma-Aldrich, Jerusalem, Israel) was determined by the eosin dye exclusion method [31].

### 2.5. Measurement of Cytopathic Activity

Cytopathic activity was assayed against HeLa cells (a kind gift from T. Kleinberger, Faculty of Medicine, Technion) (using a previously described protocol [32].

### 2.6. RNA Extraction

Total RNA was extracted from control trophozoites (WT) and AFAT using a TRI reagent kit, according to the manufacturer instructions (Sigma-Aldrich, Jerusalem, Israel).

### 2.7. RNA Sequencing (RNAseq): Library Preparation and Data Generation

Six RNAseq libraries were produced according to the manufacturer’s protocol (NEBNext UltraII Directional RNA Library Prep Kit, Illumina, NEB, MA, USA) using 800 ng of total RNA. mRNA pull-up was performed using a Magnetic Isolation Module (NEB, MA, USA). All libraries were mixed in a single tube with equal molarity. The RNAseq data was generated on an Illumina NextSeq500, 75 single-end read, high output mode (Illumina). Quality control was assessed using Fastqc (v0.11.5); reads were trimmed for adapters, low quality 3′, and minimum length of 20 using CUTADAPT (v1.12). STAR aligner (v2.6.0a) was used to align 83 bp single-end reads to an *E. histolytica* reference genome (Entamoeba_histolytica.JCVI-ESG2-1.0.dna.toplevel.fa) and annotation file (Entamoeba_histolytica.JCVI-ESG2-1.0.46.gff3), both downloaded from ENSEMBL (strain HM-1:IMSS, imported from the AmoebaDB (https://amoebadb.org/amoeba/app accessed on 28 July 2021)). The number of reads per gene was counted using Htseq-count (v0.9.1) (parameters: -t CDS -i ID -m intersection-nonempty -s reverse).

### 2.8. Descriptive Analysis

The statistical analysis was preformed using DESeq2 R package (version 1.20.0) [33].

### 2.9. Differential Expression Analysis

Results of the statistical analysis, i.e., the list of the differentially expressed genes (DEGs) (*p*-value adjusted (padj) < 0.01) are provided in the DESeq2_results_with_anno.xls file (Table S1). Genes with a fold change >1.5 were taken into account for further bioinformatics analysis. Gene symbol and gene name identification was achieved using Protein ANalysis THrough Evolutionary Relationship (PANTHER) Classification System software (http://www.pantherdb.org/ accessed on 28 July 2021) [34]. 

### 2.10. Availability of Data

RNA-Seq data are available at the Gene Expression Omnibus (http://www.ncbi.nlm.nih.gov/geo accessed on 28 July 2021) under the accession number GSE178520.

### 2.11. Construction of HA-Tagged EhTrxR Trophozoites

For the construction of the pJST4-EhTrxR expression vector that was used to express HA-tagged EhTrxR in the parasite, EhTrxR was amplified from *E. histolytica*’s genomic DNA using the primers 5′EhTrxR_KpnI (ggtaccatgagtaatattcatgatg) and 3′EhTrxR_BamHI (ggatccatgagtttgaagcc). The resulting PCR product was cloned into the pGEM-T Easy vector system (Promega, WI, USA) and then digested with the restriction enzymes, KpnI and BamHI. The digested DNA insert was subcloned into the *E. histolytica* expression vector pJST4, which was previously linearized with KpnI and BamHI. The pJST4 expression vector contains a tandem affinity purification tag for use in protein purification and identification [35]. This CHH tag contains the calmodulin binding protein, hemagglutinin (HA), and histidine (His) residues, and its expression is driven by an actin promoter.

### 2.12. Immunodetection of (HA)-Tagged EhTrxR 

*E. histolytica* control and HA-tagged EhTrxR trophozoite cytosolic proteins (40 μg) were prepared according to a published method [36] and resolved on a 10% SDS-PAGE in SDS-PAGE running buffer (25 mM Tris, 192 mM glycine, 0.1% SDS). The resultant protein bands were visualized after staining with Ponceau-S (Sigma-Aldrich, USA). Next, proteins were electrotransferred in protein transfer buffer (25 mM Tris, 192 mM glycine, 20% methanol, pH 8.3) to nitrocellulose membranes (Whatman, Protran BA83). The blots were first blocked using 3% skim milk and then probed with 1:500 mouse monoclonal HA antibody clone 12CA5 (a kind gift from Prof. Ami Aronheim) for 16 h at 4 °C. After incubation with the primary antibody, the blots were incubated with 1:5000 secondary antibody for one hour at RT (Jackson ImmunoResearch, PA, USA), and then developed using enhanced chemiluminescence (Bio RAD, Rishon Le Zion, Israel).

### 2.13. Viability Assay

*E. histolytica* trophozoite controls and EhTrxR overexpressing trophozoites (2.5 × 10^4^) were cultivated or not in the presence of 2 µM AF for 24 h. The cells were harvested at 400× *g* for 5 min, stained with Propidium iodide (1 µg/mL), and analyzed by flow cytometry. Flow cytometry was performed using Cyan ADP (Agilent Dako, CA, USA) and data from 10,000 cells were collected for each condition.

### 2.14. Detection of ROS 

WT trophozoites, AFAT, and WT trophozoites that were cultivated with AF (2 µM) for 24 h (WT + AF acute) were incubated with 0.4 mM H2DCFDA for 15 min at 37 °C. The trophozoites were washed twice with PBS, and the level of oxidation was analyzed by flow cytometry. Flow cytometry was performed using Cyan ADP flow cytometer (Agilent Dako, CA, USA) and data from 10,000 cells were collected for each condition. 

### 2.15. Detection of OXs by RAC (OX-RAC)

The detection of OXs by OX-RAC was performed using a previously described protocol [31]. A protein was considered to be oxidized when its relative amount in the DTT-treated lysates was at least two times greater than that in the untreated lysates (*p* < 0.05 according to the results of an unpaired *t*-test).

### 2.16. In-Gel Proteolysis and MS Analysis

In-gel proteolysis, MS, and data analysis were performed according to a previously reported protocol [31,37].

### 2.17. Classification of OXs According to Their Protein Class

The OXs were classified according to their protein class using PANTHER Classification System software (http://www.pantherdb.org/ accessed on 28 July 2021) [34].

### 2.18. Immunofluorescence Microscopy Analysis

The formation of F-actin in WT trophozoites, acute AF trophozoites, and AFAT was determined as described previously [28].

## 3. Results

### 3.1. Generation of AFAT

Resistance to 2 µM of AF in the parasite *T. gondi* can be generated by exposure of the parasite to the mutagenic compound *N*-Ethyl-*N*-nitrosourea [29]. To the best of our knowledge, the development by natural selection of parasites resistant to AF has never been attempted. To address this knowledge gap, we adapted *E. histolytica* trophozoites to AF by progressively increasing the drug concentration over a period of one month to 2 µM. To check whether adaptation to AF has an effect on the growth of the parasite, we compared the doubling time of AF-adapted trophozoites (AFAT) with that of wild type (WT) trophozoites. We found that the doubling time of AFAT (13 ± 0.32 h) was significantly higher than the doubling time of WT trophozoites (10.6 ± 0.24 h).

### 3.2. Reponse of AFAT to OS, NS, and Cytopathic Activity

**Figure 1:**
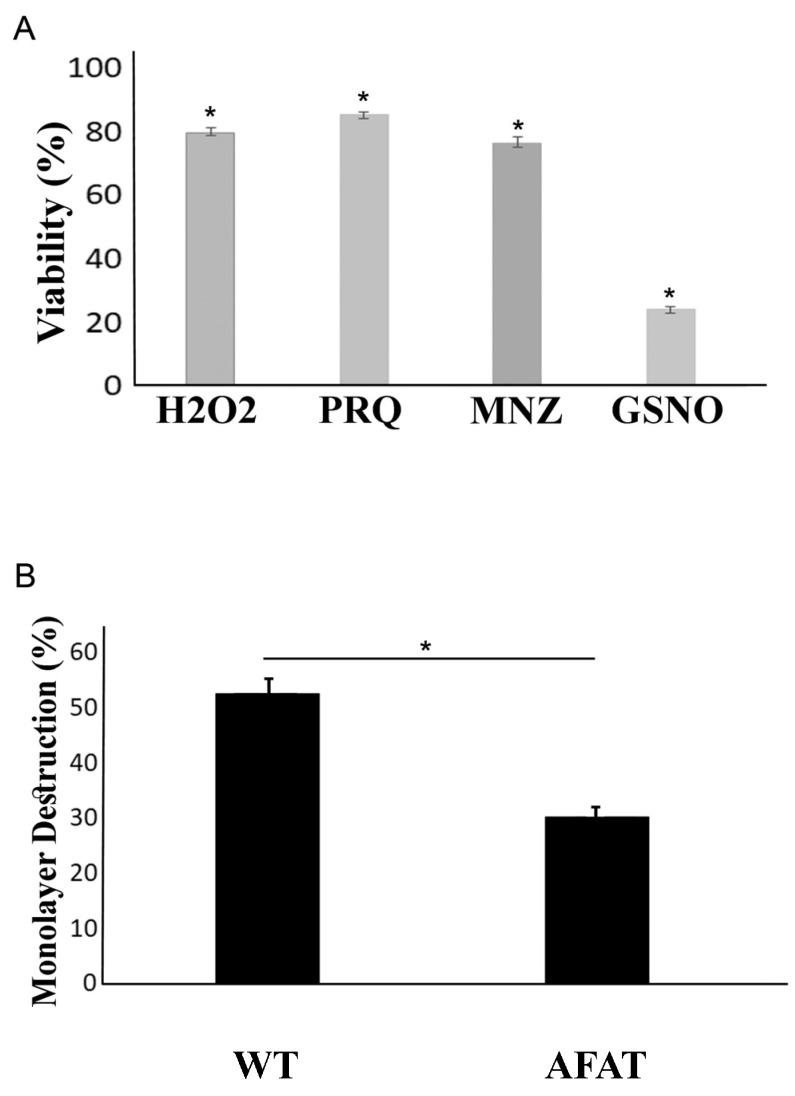
(**A**) Viability of AFAT exposed to H_2_O_2_, paraquat, MNZ, or GSNO. WT and AFAT were exposed to 2.5 mM H_2_O_2_ for 30 min, 2.5 mM paraquat (PRQ) and 5 µM metronidazole (MNZ) for 24 h, or 350 µM GSNO for 2 h. All experiments were undertaken at 37 °C. Data are expressed as the mean ± standard deviation of three independent experiments that were performed in triplicate. The graph represents the ratio percentage of viable amoebas compared to WT. The viability of AFAT exposed to H_2_O_2_, PRQ, MNZ, or GSNO was significantly different (* *p* < 0.05) from that of the WT according to the results of an unpaired Student’s *t* test. (**B**) Cytopathic activity of AFAT. Data are displayed as the mean ± standard deviation of four independent experiments that were performed in triplicate. The cytopathic activity of AFAT was significantly different (* *p* < 0.05) from that of the WT according to the results of an unpaired Student’s *t* test.

The response of AFAT to OS was tested by exposing them to H_2_O_2_ (2.5 mM for 30 min) or to paraquat (2.5 mM for 12 h). We observed that AFAT are significantly more sensitive to H_2_O_2_ or to paraquat than WT trophozoites (Figure 1A). We also examined the resistance of AFAT to MNZ (5 µM for 24 h) and found that AFAT are significantly more sensitive to MNZ than WT trophozoites (Figure 1A). The sensitivity of AFAT to nitrosative stress (NS) was tested by exposing them to the NO donor S-nitrosoglutathion (GSNO) (350 µM for 2 h). We observed that AFAT are significantly more sensitive to NS than WT trophozoites (Figure 1A). The ability of AFAT to destroy a monolayer of mammalian cells (cytopathic activity) was also determined (Figure 1B). We observed that the cytopathic activity of AFAT is impaired compared to that of WT trophozoites. Overall, these results indicate that, for *E. histolytica* trophozoites, adaptation to AF results in a loss of fitness.

### 3.3. Transcriptomics of AFAT

We used RNA sequencing (RNA-seq) to examine the mechanism of adaptation to AF. Transcriptomics of WT trophozoites vs. AFAT was compared. Our comparisons revealed that adaptation to AF has a strong effect on the *E. histolytica* transcriptome, with more than 500 upregulated and downregulated genes (Table S1).

### 3.4. Gene Categories Modulated in AFAT

**Figure 2:**
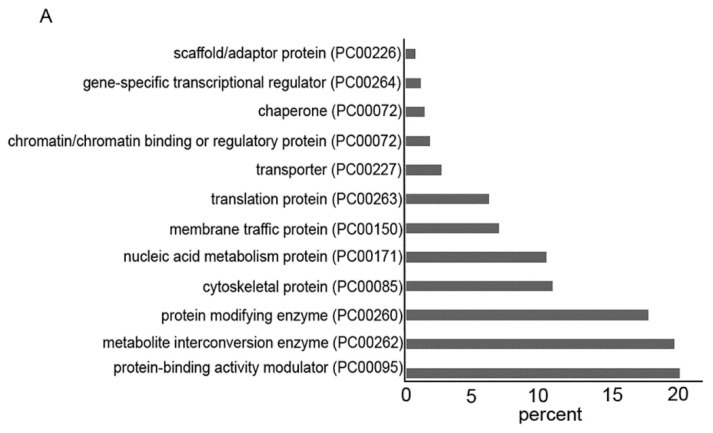

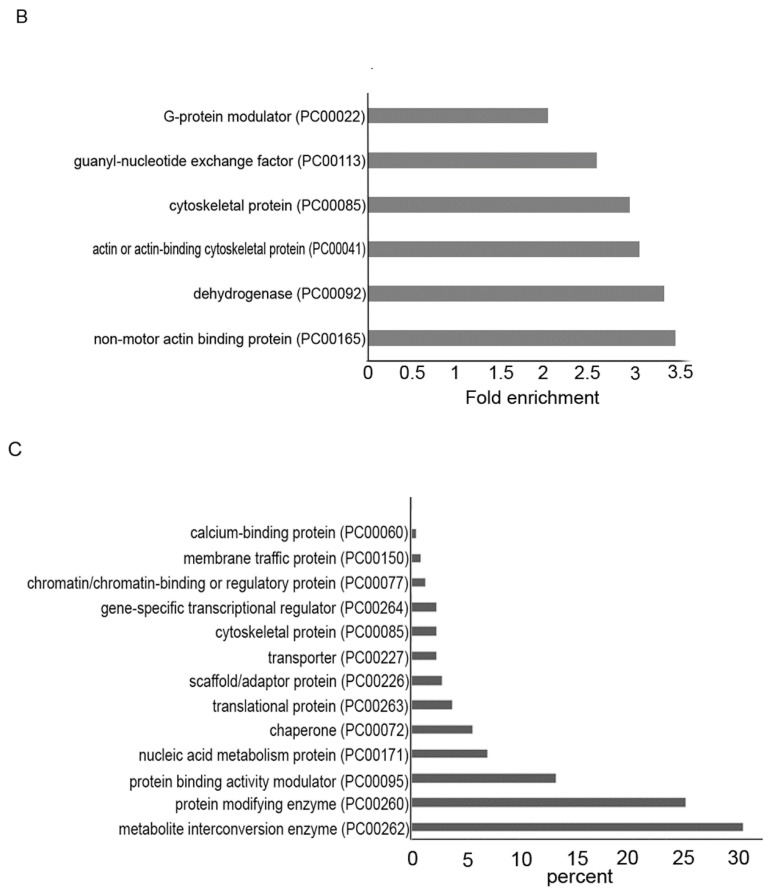
(**A**) PANTHER sequence classification of genes upregulated in AFAT; *(***B**) PANTHER statistical overrepresentation test of upregulated genes in AFAT; (**C**) PANTHER sequence classification of genes downregulated in AFAT.

The differentially regulated genes in AFAT vs. WT trophozoites were classified, according to the protein class they encode, using PANTHER. The categories for functional classification of genes upregulated in AFAT are shown in Figure 2A. The most abundant classes are the gene-encoded protein-binding activity modulator (PC00095), such as AIG1-type G domain-containing protein (EHI_176590); metabolite interconversion enzyme (PC00262), such as Lecithin:cholesterol acyltransferase (EHI_065250); protein modifying enzyme (PC00260), such as Leucine rich repeat protein phosphatase 2C domain containing protein (EHI_178020); and cytoskeletal protein (PC00085), such as F-actin-capping protein subunit beta (EHI_134490). Of the upregulated genes in AFAT, genes that encode for actin or for actin-binding cytoskeletal proteins, such as actin (EHI_107290) or EHI_172960 (Actin-related protein 2/3 complex subunit 3); dehydrogenase (PC00092), such as NAD (FAD)-dependent dehydrogenase (EHI_099700) or Aldehyde-alcohol dehydrogenase 2 (EHI_024240); and guanyl-nucleotide exchange factor, such as Ras guanine nucleotide exchange factor (EHI_023270) or Rho guanine nucleotide exchange factor (EHI_005910) are significantly enriched according to the PANTHER statistical overrepresentation test (Figure 2B).

The categories for functional classification of genes downregulated in AFAT are shown in Figure 2C. The most abundant class of gene encoded proteins are metabolite interconversion enzyme (PC00262), such as alpha-amylase (EHI_152880); protein modifying enzyme (PC00260), such as Gal/GalNAc lectin Igl2 (EHI_183000); and protein-binding activity modulator (PC00095); such as guanylate binding protein (EHI_175080). Of the downregulated genes in AFAT, no enrichment of a specific biological process was detected according to the PANTHER statistical overrepresentation test.

### 3.5. Redoxomics of AFAT

**Figure 3:**
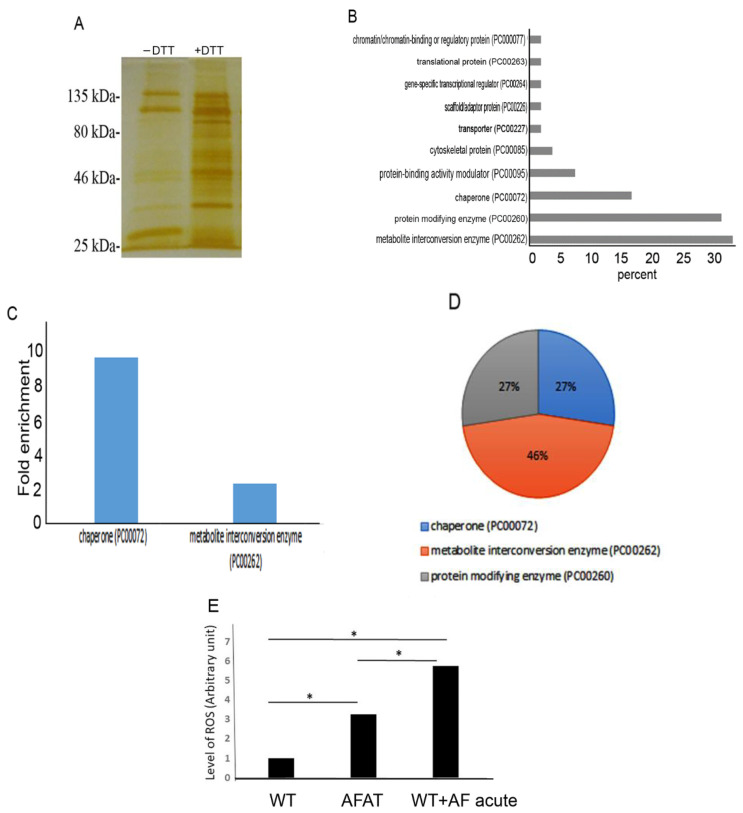
Detection of oxidized proteins by resin-assisted capture (OX-RAC) analysis of AFAT. (**A**) Silver staining of OXs. OXs in the AFAT lysates were subjected to RAC in the presence of 10 mM DTT (+DTT) or the absence of DTT (−DTT). (**B**) Protein ANalysis THrough Evolutionary Relationships (PANTHER) sequence classification of the OXs identified in AFAT. (**C**) PANTHER statistical overrepresentation test of the OXs identified in AFAT. (**D**) PANTHER sequence classification of the 17 OXs common between trophozoites exposed to an acute AF treatment [28] and AFAT. (**E**) Level of ROS in AFAT and acute AF trophozoites. WT trophozoites, AFAT, and WT trophozoites that were cultivated with AF (2 µM) for 24 h (WT + AF acute) were incubated with 0.4 mM H2DCFDA for 15 min at 37 °C. The trophozoites were washed twice with PBS, and the level of oxidation was analyzed by flow cytometry. Flow cytometry was performed using Cyan ADP (Agilent Dako, CA, USA) and data from 10,000 cells were collected for each condition. Data are expressed as the mean ± standard deviation of three independent experiments. The level of ROS in AFAT was significantly different from that of the WT + AF acute trophozoites according to the results of an unpaired Student’s *t* test (* *p* value < 0.05).

Using OX-RAC, we previously detected 583 OXs in acute AF trophozoites [28]. Here, we also used OX-RAC to detect OXs in the lysate of AFAT (Figure 3A). We identified 96 OXs in AFAT (Table S2), which were classified using PANTHER. The most abundant OX families belong to metabolite interconversion enzyme (PC00262), such as Purine nucleoside phosphorylase (EHI_200080); protein modifying enzyme (PC00260), such as NEDD8-activating enzyme E1 (EHI_098550); chaperone (PC00072), such as Peptidylprolyl isomerase (EHI_044850); and Protein-binding activity modulator (PC00095), such as glucosidase 2 subunit beta (EHI_135420) (Figure 3B). 

Of the OXs in AFAT (Table S2), chaperone (PC00072), such as HSP16 (EHI_125830) or Trx (EHI_110350), and metabolite interconversion enzyme (PC00262), such as Aminotran_5 domain-containing protein EhnifS (EHI_136380) or alpha-amylase EHI_152880, are significantly enriched according to the PANTHER statistical overrepresentation test (Figure 3C).

Seventeen OXs are shared between acute AF trophozoites [28] and AFAT (Table S3). These common OXs belong to chaperone (PC00072), metabolite interconversion enzyme (PC00262), and protein modifying enzyme (PC00260) (Figure 3D).

### 3.6. Level of ROS in AFAT

The lower quantity of OXs in AFAT compared to the quantity of OXs in acute AF trophozoites [28] suggests that AFAT are less exposed to ROS. Consequently, we measured the level of ROS with dichloro-fluorescein (H_2_DCDFC) in acute AF trophozoites and in AFAT. We observed that the ROS level in AFAT is significantly lower than that in acute AF trophozoites (Figure 3E).

### 3.7. Comparison between Transcriptomics and Redoxomics of AFAT

We found that only two genes upregulated in AFAT (Gal/GalNAc lectin Igl1 EHI_006980 and SNF7 family protein EHI_077530) have their product oxidized (Table S3). None of the genes downregulated in AFAT have their product oxidized (Table S3).

### 3.8. Comparison between Transcriptomics of AFAT and Redoxomics of Acute AF Trophozoites

**Figure 4:**
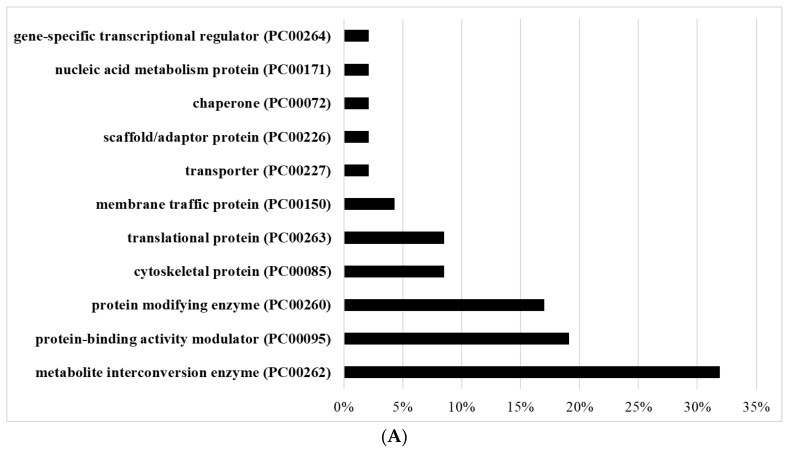

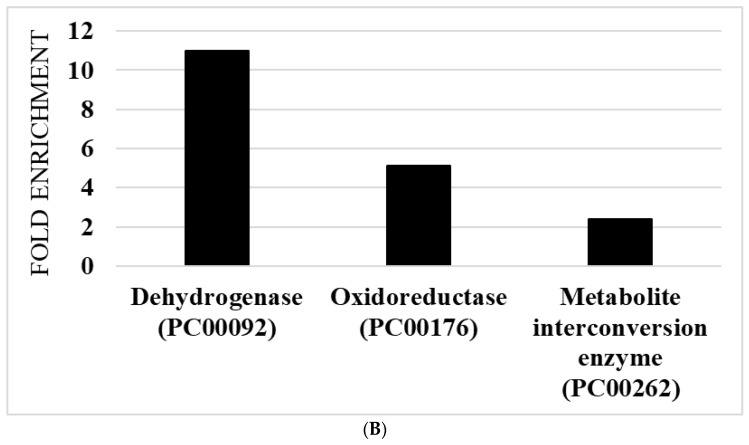
Comparison between transcriptomics of AFAT and redoxomics of acute AF trophozoites. (**A**) PANTHER sequence classification of genes upregulated in AFAT that have their product oxidized in acute AF trophozoites. (**B**) PANTHER statistical overrepresentation test of upregulated genes in AFAT that have their product oxidized in acute AF trophozoites.

We found that 77 genes upregulated in AFAT have their product oxidized in acute AF trophozoites [28] (Table S3). The most abundant OXs belong to metabolite interconversion enzyme (PC00262), protein-binding activity modulator (PC00095), protein modifying enzyme (PC00260), and cytoskeletal protein (PC00085) (Figure 4A). Of the upregulated genes in AFAT that have their product oxidized in acute AF trophozoites, genes that encode for dehydrogenase (PC00092), such as NAD(FAD)-dependent dehydrogenase (EHI_099700); oxydoreductase (PC00176), such as Pyruvate:ferredoxin oxidoreductase (EHI_051060); and metabolite interconversion enzymes (PC00262), such as isopentenyl phosphate kinase (EHI_178490), are significantly enriched according to the PANTHER statistical overrepresentation test (Figure 4B).

Eight genes that are downregulated in AFAT have their product oxidized in acute AF trophozoites [28] (Table S3). These OXs are the uncharacterized proteins (EHI_008120, EHI_065710, and EHI_110780), Asparagine--tRNA ligase (EHI_126920), Cytosolic Fe-S cluster assembly factor NUBP1 (EHI_047750), ribonuclease (EHI_156310), and Flavodoxin-like domain-containing protein (EHI_096710).

### 3.9. Formation of F-Actin in AFAT

**Figure 5:**
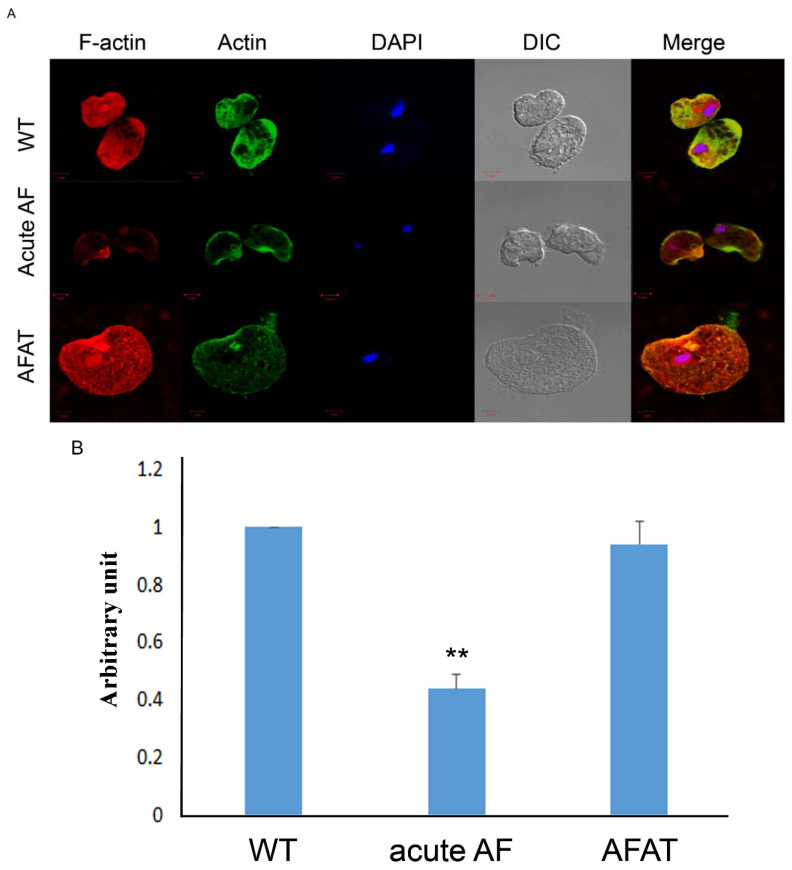
Formation of F-actin in WT trophozoites, acute AF trophozoites, and AFAT. (**A**) Confocal laser scanning microscopy of F-actin and total actin in WT trophozoites, acute AF trophozoites, and AFAT showed that F-actin was detected using rhodamine-conjugated phalloidin. Total actin was detected using a primary actin antibody and a secondary Cy2-conjugated immunoglobulin G (IgG) antibody. The nuclei (blue) were stained by 4′,6-diamidino-2-phenylindole (DAPI). (**B**) A computer-assisted image was overlaid on the signal emitted by the actin antibody, phalloidin, and DAPI. Fluorescence quantification was performed using Fiji software [38] on 10 trophozoites and the F-actin signal was normalized to the total actin signal. The level of F-actin in WT was arbitrary defined as 1. Data are expressed as the mean ± standard deviation of two independent experiments. The level of F-actin in acute AF trophozoites was significantly different from that in WT and AFAT according to the results of an unpaired Student’s *t* test (** *p* value < 0.01). No difference of F-actin level between WT and AFAT was observed according to the results of an unpaired Student’s *t* test (*p* value > 0.05).

We have previously shown that AF leads to the oxidation of cytoskeletal proteins and inhibits the formation of actin filaments (F-actin) [28]. In contrast, cytoskeletal proteins in AFAT are not significantly enriched among OXs according to the PANTHER statistical overrepresentation test (Table S2). In order to confirm this observation, we looked at the level of F-actin in WT trophozoites, acute AF trophozoites, and AFAT. As described previously [28], the F-actin signal in acute AF trophozoites was significantly less intense than that in WT trophozoites. In contrast, the F-actin signal was identical in WT trophozoites and AFAT (Figure 5A,B). These results confirm that the formation of F-actin is impaired in acute AF trophozoites [28], but is not impaired in AFAT.

### 3.10. Overexpression of EhTrxR Does Not Protect E. histolytica Trophozoites against AF

**Figure 6:**
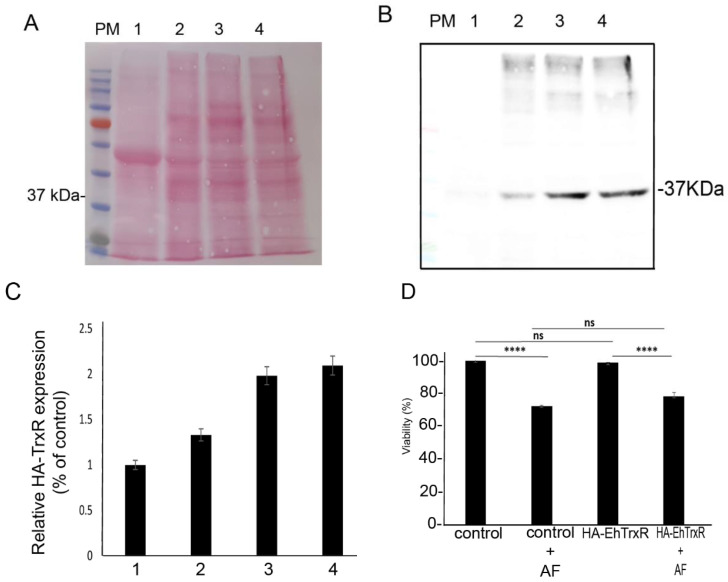
Western blot analysis of *E. histolytica* trophozoites that overexpress a hemagglutinin (HA)-tagged EhTrxR and viability assay. Legend: Protein molecular weight marker (PM). Control trophozoites (lane 1). HA-tagged EhTrxR trophozoites cultivated in the presence of an increasing concentration of G418 (lane 2: 6 µg/mL, lane 3: 30 µg/mL, lane 4: 48 µg/mL). (**A**) Ponceau staining of a nitrocellulose membrane containing cytosolic proteins (40 µg) separated by SDS PAGE of control trophozoites and of HA-tagged EhTrxR trophozoites cultivated in the presence of an increasing concentration of G418. (**B**) Immunodetection of (HA)-tagged EHTrxR with an HA monoclonal antibody (1:500) in HA-tagged EhTrxR trophozoites cultivated in the presence of an increasing concentration of G418. (**C**) Relative quantification of the HA EhTrxR signal following its normalization with the level of total protein in each well with ImageJ software. Normalized values for control trophozoites were taken as 100%. These results are representative of two independent experiments. (**D**) Viability assay. *E. histolytica* trophozoite controls and HA-tagged EhTrxR trophozoites were cultivated in the presence of 2 µM AF for 24 h. The cells were harvested at 400× *g* for 5 min, stained with Propidium iodide, and analyzed by flow cytometry. Flow cytometry was performed using Cyan ADP (Agilent Dako, CA, USA) and data from 10,000 cells were collected for each condition. Data are expressed as the mean ± standard deviation of three independent experiments that were performed in triplicate. The viability of the control trophozoites was defined as 100%. The viability of control trophozoites was not significantly different (ns) from that of the HA-tagged EhTrxR trophozoites according to the results of an unpaired Student’s *t* test (*p* value < 0.05). The viability of control trophozoites was significantly different from that of the control trophozoites exposed to AF according to the results of an unpaired Student’s *t* test (**** *p* value < 0.001). The viability of HA-tagged EhTrxR trophozoites was significantly different from that of the HA-tagged EhTrxR trophozoites exposed to AF according to the results of an unpaired Student’s *t* test (**** *p* value < 0.001).

Overexpression of TrxR in the parasite *Giardia lamblia* has no effect on its resistance to AF [26]. In *E. histolytica*, Debnath et al. found that AF inhibits the amebic TrxR and its reduction, leading to a higher sensitivity of trophozoites to ROS-mediated killing [39]. Our observations regarding the level of TrxR expression, which was the same in WT trophozoites and in AFAT (Table S1), and the fact that Trxs are enriched OXs in AFAT, strongly suggest that *E. histolytica* TrxR is not central to the mechanism of adaptation of the parasite to AF. To test this hypothesis, we overexpressed EhTrxR in *E. histolytica* trophozoites. Overexpression of EhTrxR was confirmed by Western blotting and its level of expression in *E. histolytica* was proportional to the amount of G418 used for selection (Figure 6A–C) [40]. Next, we determined the level of resistance to AF of HA-tagged EhTrxR trophozoites. We observed that the level of resistance to AF of HA-tagged EhTrxR trophozoites did not differ significantly from the level of resistance of the control trophozoites (trophozoites transfected with pEhExGFP (a kind gift from Dr. Tomoyoshi Nozaki [41])) (Figure 6D). pEhExGFP allows the constitutive expression of the green fluorescent protein (GFP).

## 4. Discussion

In our previous work, we demonstrated that AF triggers OS inside *E. histolytica* trophozoites, resulting in the oxidation of more than 500 proteins, including many redox enzymes that are essential for controlling the intracellular levels of ROS in the parasite [28,42,43]. Here, we characterized *E. histolytica* trophozoites that were adapted to 2 µM AF. Adaptation of *E. histolytica* to AF leads to the upregulation and downregulation of hundreds of genes, which suggests that the mechanism of adaptation is complex. Drug resistance is often mediated by a drug’s molecular target gene overexpression [44,45]. Consequently, we expected that *E. histolytica* TrxR (EhTrxR), the assumed main target of AF [25], would be one of the upregulated genes in AFAT. However, transcriptomics of AFAT indicates that this was not the case. Indeed, the overexpression of EhTrxR did not confer to *E. histolytica* resistance to AF. This information raises a question about why EhTrxR expression is not upregulated as a simple mechanism to resist AF. One possible answer is that, as for *Giardia lamblia*, TrxR is not the primary target of AF in *E. histolytica* [26]. This is supported by the absence of the detection of EhTrxR among OXs in AFAT (this work) and acute AF trophozoites [28].

It is also possible that the fitness cost for *E. histolytica* to overexpress TrxR during adaptation to AF resistance is too high. EhTrxR can generate H_2_O_2_ from molecular oxygen, leading to the formation of reactive species [46]. Therefore, it is possible that the production of H_2_O_2_ resulting from EhTrxR overexpression combined with OS triggered by AF [28] during the adaptation process cannot be tolerated by the parasite.

In this work, we found that only two genes upregulated in AFAT have their products oxidized in AFAT. In contrast, 77 genes upregulated in AFAT have their product oxidized in acute AF trophozoites [28]. The upregulation of these 77 genes in AFAT may be essential for the adaption of the parasite to AF by replacing their oxidized-inactivated products by reduced-activated proteins. The relevance of this mechanism for some of these 77 genes is discussed in the following.

Pyruvate:ferredoxin oxidoreductase (EHI_051060), NADP-dependent alcohol dehydrogenase (EHI_107210), and Fe-ADH domain-containing protein (EHI_198760), which encode for proteins involved in redox regulation: These redox enzymes depend on cysteine residues for their activity [47,48,49]. The oxidation of these cysteine residues impairs their activity [47,50].

Genes that encode the protein-binding activity modulator, such as Ras guanine nucleotide exchange factor (EHI_035800), Rho guanine nucleotide exchange factor (EHI_005910), or Ras GTPase-activating protein (EHI_105250): These proteins have their product oxidized in acute AF trophozoites [28]. G proteins are involved in vesicular trafficking and cytoskeleton regulation [51]. Redox regulation of G-proteins have been well documented [52] and their oxidation impairs *E. histolytica*’s motility [28].

Genes that encode protein-modifying enzymes such as protein kinase domain-containing proteins (EHI_186820) (EHI_101280) and Protein kinase (EHI_188110), which are also oxidized in acute AF trophozoites [28]: Protein kinases have been associated with the virulence and phagocytic activity of *E. histolytica* [53]. The redox regulation of protein kinases is well established [54], and it has been demonstrated that AF can directly inhibit protein kinase C by interacting with thiol groups present in the catalytic site [55].

Genes that encode actin or actin-binding cytoskeletal proteins are upregulated in AFAT and oxidized in acute AF trophozoites [28]: In our previous work, we showed that AF induces the oxidation of *E. histolytica* cytoskeletal proteins and consequently inhibits the formation of F-actin [28]. Consequently, it appears that the parasite upregulated the expression of actin-binding cytoskeletal proteins as a mechanism to adapt to AF by replacing oxidized cytoskeletal proteins that were formed during the process of adaptation to AF. The low level of F-actin in acute AF trophozoites and the normal level of F-actin in AFAT (this work) support this hypothesis.

The fact that *E. histolytica* can adapt to AF illustrates the remarkable ability of *E. histolytica* to adapt to drugs [56,57] and environmental stresses [32,58]. The fitness cost paid by the parasite to adapt to AF resembles collateral sensitivity, which occurs when the acquisition of resistance to one antibiotic produces increased susceptibility to a second antibiotic [59]. AFAT are more sensitive to OS, paraquat, MNZ, and GSNO than WT trophozoites. Resistance to OS in *E. histolytica* involves the upregulation of 29 kDa peroxiredoxin [60] and iron-containing peroxide dismutase expression, which is also involved in the resistance to MNZ [10,61]. The level of expression of 29 kDa peroxiredoxin and iron-containing peroxide dismutase is globally the same in WT and in AFAT, which suggests that the sensitivity of AFAT to OS and MNZ is not caused by a reduced level of these redox enzymes’ expressions. As discussed above, many oxidized proteins in AFAT have their level of expression upregulated. The fitness cost observed in AFAT may be due to numerous factors, including the rerouting of protein synthesis toward oxidized proteins, or substrate wasting that results from target overexpression [62]. In hydroxamic acid analog pan-histone deacetylase inhibitor-resistant leukemia cells, overexpression of the target protein heat shock protein 90 (HSP90) revealed collateral sensitivity to the HSP90 inhibitor 17-*N*-allylamino-17-demethoxygeldanamycin [63].

## 5. Conclusions

We showed that *E. histolytica* trophozoites can be easily selected to resist toxic concentrations of AF in vitro. Adaptation to AF reduces the fitness of *E. histolytica*, as seen in a decreased growth rate and virulence, and a sensitivity to OS, NS, and MNZ. Overexpression of genes whose products are sensitive to AF-mediated oxidation may represent an important step in the adaptation process to AF, and EhTrxR does not appear to be central to this process.

AF is FDA approved for the treatment of rheumatoid arthritis but has not been yet used as an antimicrobial drug in the field. The ability of *E. histolytica* to adapt to amebicidal concentrations of AF raises concerns about the future use of this drug as an antiamebic compound. Our omics data provide the basis for the development of strategies to limit the emergence of resistance against AF. One possible strategy suggested by our data is to promote dual antibiotic therapy (AF + MNZ) vs. single AF therapy, because adaptation to AF leads to more MNZ sensitivity in *E. histolytica*.

## Data Availability

RNA-Seq data have been deposited at the Gene Expression Omnibus (http://www.ncbi.nlm.nih.gov/geo accessed on 28 July 2021) under the accession number GSE178520.

## Supplementary Materials

The following are available online at https://www.mdpi.com/article/10.3390/antiox10081240/s1. Table S1: transcriptomics of WT trophozoites vs. AFAT. Table S2: legend of Table S1. Table S3: comparative analysis of OXs in AFAT vs. OXs in acute AF; OXs in AFAT vs. gene products upregulated in AFAT and OXs in acute AF vs. gene products upregulated or downregulated in AFAT.

## References

[1] Turkeltaub J.A., McCarty T.R., Hotez P.J. (2015). The intestinal protozoa: Emerging impact on global health and development. Curr. Opin. Gastroenterol..

[2] Powell S.J., MacLeod I., Wilmot A.J., Elsdon-Dew R. (1966). Metronidazole in amoebic dysentery and amoebic liver abscess. Lancet.

[3] Leitsch D., Kolarich D., Binder M., Wilson I.B.H., Altmann F., Duchene M. (2007). Nitroimidazole action in *Entamoeba histolytica*: A central role for thioredoxin reductase. PLoS Biol..

[4] Cowdrey S.C. (1975). Letter: Hazards of metronidazole. N. Engl. J. Med..

[5] Andersson K.E. (1981). Pharmacokinetics of Nitroimidazoles-Spectrum of Adverse Reactions. Scand. J. Infect. Dis. Suppl..

[6] Roe F.J. (1977). Metronidazole: Review of uses and toxicity. J. Antimicrob. Chemother..

[7] Camacho N., Espinoza C., Rodriguez C., Rodriguez E. (2008). Isolates of Clostridium perfringens recovered from Costa Rican patients with antibiotic-associated diarrhoea are mostly enterotoxin-negative and susceptible to first-choice antimicrobials. J. Med. Microbiol..

[8] Hashemi S.J., Sheikh A.F., Goodarzi H., Yadyad M.J., Seyedian S.S., Aslani S. (2019). Genetic basis for metronidazole and clarithromycin resistance in Helicobacter pylori strains isolated from patients with gastroduodenal disorders. Infect. Drug Resist..

[9] Wassmann C., Bruchhaus I. (2000). Superoxide dismutase reduces susceptibility to metronidazole of the pathogenic protozoan *Entamoeba histolytica* under microaerophilic but not under anaerobic conditions. Arch. Biochem. Biophys..

[10] Wassmann C., Hellberg A., Tannich E., Bruchhaus I. (1999). Metronidazole resistance in the protozoan parasite Entamoeba *histolytica* is associated with increased expression of iron-containing superoxide dismutase and peroxiredoxin and decreased expression of ferredoxin 1 and flavin reductase. J. Biol. Chem..

[11] Upcroft J.A., Upcroft P. (2001). Drug susceptibility testing of anaerobic protozoa. Antimicrob. Agents Chemother..

[12] Finkelstein A.E., Walz D.T., Batista V., Mizraji M., Roisman F., Misher A. (1976). Auranofin. New oral gold compound for treatment of rheumatoid arthritis. Ann. Rheum. Dis..

[13] Gromer S., Arscott L.D., Williams C.H., Schirmer R.H., Becker K. (1998). Human placenta thioredoxin reductase. Isolation of the selenoenzyme, steady state kinetics, and inhibition by therapeutic gold compounds. J. Biol. Chem..

[14] Onodera T., Momose I., Kawada M. (2019). Potential Anticancer Activity of Auranofin. Chem. Pharm. Bull..

[15] Ruth M.M., van Rossum M., Koeken V., Pennings L.J., Svensson E.M., Ruesen C. (2019). Auranofin Activity Exposes Thioredoxin Reductase as a Viable Drug Target in Mycobacterium abscessus. Antimicrob. Agents Chemother..

[16] AbdelKhalek A., Abutaleb N.S., Mohammad H., Seleem M.N. (2019). Antibacterial and antivirulence activities of auranofin against Clostridium difficile. Int. J. Antimicrob. Agents.

[17] Jackson-Rosario S., Cowart D., Myers A., Tarrien R., Levine R.L., Scott R.A. (2009). Auranofin disrupts selenium metabolism in Clostridium difficile by forming a stable Au-Se adduct. J. Biol. Inorg. Chem..

[18] Abutaleb N.S., Seleem M.N. (2020). Antivirulence activity of auranofin against vancomycin-resistant enterococci: In vitro and in vivo studies. Int. J. Antimicrob. Agents.

[19] AbdelKhalek A., Abutaleb N.S., Elmagarmid K.A., Seleem M.N. (2018). Repurposing auranofin as an intestinal decolonizing agent for vancomycin-resistant enterococci. Sci. Rep..

[20] Thangamani S., Mohammad H., Abushahba M.F., Sobreira T.J., Hedrick V.E., Paul L.N. (2016). Antibacterial activity and mechanism of action of auranofin against multi-drug resistant bacterial pathogens. Sci. Rep..

[21] Angelucci F., Sayed A.A., Williams D.L., Boumis G., Brunori M., Dimastrogiovanni D. (2009). Inhibition of Schistosoma mansoni thioredoxin-glutathione reductase by auranofin: Structural and kinetic aspects. J. Biol. Chem..

[22] Kuntz A.N., Davioud-Charvet E., Sayed A.A., Califf L.L., Dessolin J., Arner E.S. (2007). Thioredoxin glutathione reductase from Schistosoma mansoni: An essential parasite enzyme and a key drug target. PLoS Med..

[23] Hopper M., Yun J.F., Zhou B., Le C., Kehoe K., Le R. (2016). Auranofin inactivates Trichomonas vaginalis thioredoxin reductase and is effective against trichomonads in vitro and in vivo. Int. J. Antimicrob. Agents.

[24] Tejman-Yarden N., Miyamoto Y., Leitsch D., Santini J., Debnath A., Gut J. (2013). A reprofiled drug, auranofin, is effective against metronidazole-resistant Giardia lamblia. Antimicrob. Agents Chemother..

[25] Debnath A., Parsonage D., Andrade R.M., He C., Cobo E.R., Hirata K. (2012). A high-throughput drug screen for *Entamoeba histolytica* identifies a new lead and target. Nat. Med..

[26] Leitsch D., Muller J., Muller N. (2016). Evaluation of Giardia lamblia thioredoxin reductase as drug activating enzyme and as drug target. Int. J. Parasitol. Drugs Drug Resist..

[27] Mi-Ichi F., Ishikawa T., Tam V.K., Deloer S., Hamano S., Hamada T., Yoshida H. (2019). Characterization of *Entamoeba histolytica* adenosine 5’-phosphosulfate (APS) kinase; validation as a target and provision of leads for the development of new drugs against amoebiasis. PLoS Negl. Trop. Dis..

[28] Shaulov Y., Nagaraja S., Sarid L., Trebicz-Geffen M., Ankri S. (2020). Formation of oxidised (OX) proteins in *Entamoeba histolytica* exposed to auranofin and consequences on the parasite virulence. Cell. Microbiol..

[29] Ma C.I., Tirtorahardjo J.A., Jan S., Schweizer S.S., Rosario S.A.C., Du Y. (2021). Auranofin Resistance in Toxoplasma gondii Decreases the Accumulation of Reactive Oxygen Species but Does Not Target Parasite Thioredoxin Reductase. Front. Cell. Infect. Microbiol..

[30] Diamond L.S., Harlow D.R., Cunnick C.C. (1978). A new medium for the axenic cultivation of *Entamoeba histolytica* and other Entamoeba. Trans. R. Soc. Trop. Med. Hyg..

[31] Shahi P., Trebicz-Geffen M., Nagaraja S., Alterzon-Baumel S., Hertz R., Methling K. (2016). Proteomic Identification of Oxidized Proteins in *Entamoeba histolytica* by Resin-Assisted Capture: Insights into the Role of Arginase in Resistance to Oxidative Stress. PLoS Negl. Trop. Dis..

[32] Trebicz-Geffen M., Shahi P., Nagaraja S., Vanunu S., Manor S., Avrahami A., Ankri S. (2017). Identification of S-Nitrosylated (SNO) Proteins in *Entamoeba histolytica* Adapted to Nitrosative Stress: Insights into the Role of SNO Actin and In vitro Virulence. Front. Cell. Infect. Microbiol..

[33] Love M.I., Huber W., Anders S. (2014). Moderated estimation of fold change and dispersion for RNA-seq data with DESeq2. Genome Biol..

[34] Mi H., Ebert D., Muruganujan A., Mills C., Albou L.P., Mushayamaha T. (2021). PANTHER version 16: A revised family classification, tree-based classification tool, enhancer regions and extensive API. Nucleic Acids Res..

[35] Dastidar P.G., Majumder S., Lohia A. (2007). Eh Klp5 is a divergent member of the kinesin 5 family that regulates genome content and microtubular assembly in *Entamoeba histolytica*. Cell. Microbiol..

[36] Lavi T., Isakov E., Harony H., Fisher O., Siman-Tov R., Ankri S. (2006). Sensing DNA methylation in the protozoan parasite *Entamoeba histolytica*. Mol. Microbiol..

[37] Cox J., Mann M. (2008). MaxQuant enables high peptide identification rates, individualized p.p.b.-range mass accuracies and proteome-wide protein quantification. Nat. Biotechnol..

[38] Schindelin J., Arganda-Carreras I., Frise E., Kaynig V., Longair M., Pietzsch T., Preibisch S., Rueden C., Saalfeld S., Schmid B. (2012). Fiji: An open-source platform for biological-image analysis. Nat. Methods.

[39] Parsonage D., Sheng F., Hirata K., Debnath A., McKerrow J.H., Reed S.L., Abagyan R., Poole L.B., Podust L.M. (2016). X-ray structures of thioredoxin and thioredoxin reductase from *Entamoeba histolytica* and prevailing hypothesis of the mechanism of Auranofin action. J. Struct. Biol..

[40] Hamann L., Nickel R., Tannich E. (1995). Transfection and continuous expression of heterologous genes in the protozoan parasite *Entamoeba histolytica*. Proc. Natl. Acad. Sci. USA.

[41] Yousuf M.A., Mi-ichi F., Nakada-Tsukui K., Nozaki T. (2010). Localization and targeting of an unusual pyridine nucleotide transhydrogenase in *Entamoeba histolytica*. Eukaryot. Cell.

[42] Ankri S. (2021). *Entamoeba histolytica*-Gut Microbiota Interaction: More Than Meets the Eye. Microorganisms.

[43] Pineda E., Perdomo D. (2017). *Entamoeba histolytica* under Oxidative Stress: What Countermeasure Mechanisms Are in Place?. Cells.

[44] Chopra I. (1998). Over-expression of target genes as a mechanism of antibiotic resistance in bacteria. J. Antimicrob. Chemother..

[45] Capela R., Moreira R., Lopes F. (2019). An Overview of Drug Resistance in Protozoal Diseases. Int. J. Mol. Sci..

[46] Arias D.G., Regner E.L., Iglesias A.A., Guerrero S.A. (2012). *Entamoeba histolytica* thioredoxin reductase: Molecular and functional characterization of its atypical properties. Biochim. Et Biophys. Acta.

[47] Pineda E., Encalada R., Rodriguez-Zavala J.S., Olivos-Garcia A., Moreno-Sanchez R., Saavedra E. (2010). Pyruvate:ferredoxin oxidoreductase and bifunctional aldehyde-alcohol dehydrogenase are essential for energy metabolism under oxidative stress in *Entamoeba histolytica*. FEBS J..

[48] Kumar A., Shen P.S., Descoteaux S., Pohl J., Bailey G., Samuelson J. (1992). Cloning and expression of an NADP(+)-dependent alcohol dehydrogenase gene of *Entamoeba histolytica*. Proc. Natl. Acad. Sci. USA.

[49] König C., Meyer M., Lender C., Nehls S., Wallaschkowski T., Holm T., Matthies T., Lercher D., Matthiesen J., Fehling H. (2020). An Alcohol Dehydrogenase 3 (ADH3) from *Entamoeba histolytica* Is Involved in the Detoxification of Toxic Aldehydes. Microorganisms.

[50] Klomsiri C., Karplus P.A., Poole L.B. (2011). Cysteine-based redox switches in enzymes. Antioxid. Redox Signal..

[51] Bosch D.E., Siderovski D.P. (2013). G protein signaling in the parasite *Entamoeba histolytica*. Exp. Mol. Med..

[52] Accorsi K., Giglione C., Vanoni M., Parmeggiani A. (2001). The Ras GDP/GTP cycle is regulated by oxidizing agents at the level of Ras regulators and effectors. FEBS Lett..

[53] Ahmad A., Mishra S., Som L., Gourinath S. (2020). Role of kinases in virulence and pathogenesis of protozoan parasite *E. Histolytica*. Front. Biosci..

[54] Corcoran A., Cotter T.G. (2013). Redox regulation of protein kinases. FEBS J..

[55] Froscio M., Murray A.W., Hurst N.P. (1989). Inhibition of protein kinase C activity by the antirheumatic drug auranofin. Biochem. Pharmacol..

[56] Penuliar G.M., Nakada-Tsukui K., Nozaki T. (2015). Phenotypic and transcriptional profiling in *Entamoeba histolytica* reveal costs to fitness and adaptive responses associated with metronidazole resistance. Front. Microbiol..

[57] Ehrenkaufer G.M., Suresh S., Solow-Cordero D., Singh U. (2018). High-Throughput Screening of Entamoeba Identifies Compounds Which Target Both Life Cycle Stages and Which Are Effective Against Metronidazole Resistant Parasites. Front. Cell. Infect. Microbiol..

[58] Baumel-Alterzon S., Weber C., Guillen N., Ankri S. (2013). Identification of dihydropyrimidine dehydrogenase as a virulence factor essential for the survival of *Entamoeba histolytica* in glucose-poor environments. Cell. Microbiol..

[59] Herencias C., Rodriguez-Beltran J., Leon-Sampedro R., Alonso-Del Valle A., Palkovicova J., Canton R. (2021). Collateral sensitivity associated with antibiotic resistance plasmids. eLife.

[60] Tekwani B.L., Mehlotra R.K. (1999). Molecular basis of defence against oxidative stress in *Entamoeba histolytica* and Giardia lamblia. Microbes Infect.

[61] Bruchhaus I., Tannich E. (1994). Induction of the iron-containing superoxide dismutase in *Entamoeba histolytica* by a superoxide anion-generating system or by iron chelation. Mol. Biochem. Parasitol..

[62] Palmer A.C., Kishony R. (2014). Opposing effects of target overexpression reveal drug mechanisms. Nat. Commun..

[63] Fiskus W., Rao R., Fernandez P., Herger B., Yang Y., Chen J. (2008). Molecular and biologic characterization and drug sensitivity of pan-histone deacetylase inhibitor-resistant acute myeloid leukemia cells. Blood.

